# A platform trial design for preventive vaccines against Marburg virus and other emerging infectious disease threats

**DOI:** 10.1177/17407745221110880

**Published:** 2022-07-22

**Authors:** Ira M Longini, Yang Yang, Thomas R Fleming, César Muñoz-Fontela, Rui Wang, Susan S Ellenberg, George Qian, M Elizabeth Halloran, Martha Nason, Victor De Gruttola, Sabue Mulangu, Yunda Huang, Christl A Donnelly, Ana-Maria Henao Restrepo

**Affiliations:** 1 Department of Biostatistics, University of Florida, Gainesville, FL, USA; 2 Department of Biostatistics, University of Washington, Seattle, WA, USA; 3 Bernhard-Nocht-Institute for Tropical Medicine, Hamburg, Germany; 4 German Center for Infection Research, DZIF, Partner site Hamburg, Hamburg, Germany; 5 Department of Population Medicine, Harvard Pilgrim Health Care Institute and Harvard Medical School, Boston, MA, USA; 6 Department of Biostatistics, Harvard University, Boston, MA, USA; 7 Department of Biostatistics, Epidemiology, and Informatics, University of Pennsylvania, Philadelphia, PA, USA; 8 London School of Hygiene & Tropical Medicine, London, UK; 9 Vaccine and Infectious Disease Division, Fred Hutchinson Cancer Center, Seattle, WA, USA; 10 Biostatistics Research Branch, National Institute of Allergy and Infectious Diseases (NIAID/NIH), Bethesda, MD, USA; 11 Institut National de Recherche Biomédicale, Kinshasa, Democratic Republic of the Congo; 12 Department of Statistics, University of Oxford, Oxford, UK; 13 Department of Infectious Disease Epidemiology, Imperial College London, London, UK; 14 World Health Organization, Geneva, Switzerland

**Keywords:** Randomized placebo-controlled vaccine trial, cluster-randomized vaccine trial, Marburg virus, vaccine efficacy, emerging infectious disease threat

## Abstract

**Background:**

The threat of a possible Marburg virus disease outbreak in Central and Western Africa is growing. While no Marburg virus vaccines are currently available for use, several candidates are in the pipeline. Building on knowledge and experiences in the designs of vaccine efficacy trials against other pathogens, including SARS-CoV-2, we develop designs of randomized Phase 3 vaccine efficacy trials for Marburg virus vaccines.

**Methods:**

A core protocol approach will be used, allowing multiple vaccine candidates to be tested against controls. The primary objective of the trial will be to evaluate the effect of each vaccine on the rate of virologically confirmed Marburg virus disease, although Marburg infection assessed via seroconversion could be the primary objective in some cases. The overall trial design will be a mixture of individually and cluster-randomized designs, with individual randomization done whenever possible. Clusters will consist of either contacts and contacts of contacts of index cases, that is, ring vaccination, or other transmission units.

**Results:**

The primary efficacy endpoint will be analysed as a time-to-event outcome. A vaccine will be considered successful if its estimated efficacy is greater than 50% and has sufficient precision to rule out that true efficacy is less than 30%. This will require approximately 150 total endpoints, that is, cases of confirmed Marburg virus disease, per vaccine/comparator combination. Interim analyses will be conducted after 50 and after 100 events. Statistical analysis of the trial will be blended across the different types of designs. Under the assumption of a 6-month attack rate of 1% of the participants in the placebo arm for both the individually and cluster-randomized populations, the most likely sample size is about 20,000 participants per arm.

**Conclusion:**

This event-driven design takes into the account the potentially sporadic spread of Marburg virus. The proposed trial design may be applicable for other pathogens against which effective vaccines are not yet available.

## Background

The World Health Organization was notified on August 3, 2021 of a confirmed case of Marburg virus (MARV) disease in the Guéckédou prefecture in the Republic of Guinea.^1^ As this was the first time MARV disease was detected in the country, concerns were raised about a possible epidemic. Fortunately, no additional MARV disease cases were detected in the area.^2^ Nevertheless, the emergence of MARV in Guinea highlights the continued presence of filoviral diseases in Central and Western Africa, and strongly suggests that, before the rapid implementation of diagnostics and surveillance that followed the Ebola virus epidemic in West Africa in 2014,^3^ many outbreaks may have gone undetected. Predictably, since 2014, cases of filovirus disease have been seen annually. Moreover, filovirus sequences (including MARV and Bombali virus) have been identified in bats in West Africa,^4,5^ suggesting that MARV and other filoviruses are endemic in rainforest areas of Sub-Saharan Africa. Importantly, in contrast to Ebola virus, currently there are no licenced vaccines against MARV. To enhance the rapid development of MARV vaccines, the World Health Organization R&D Blueprint has convened a group of experts from industry, government and academia to share knowledge, assays, laboratory networks and animal models with the ultimate goal to promote preclinical and clinical development of MARV vaccine candidates.^6^ This World Health Organization-coordinated consortium for the development and evaluation of MARV vaccines is based on the same sharing principles that governed the scientific interactions of the World Health Organization COVID-19 working groups and that accelerated the development of COVID-19 vaccines^7^ and therapeutics.^8^ A number of Marburg virus vaccine candidates are in the pipeline. A DNA–MARV vaccine was manufactured and showed favourable safety and immunogenicity in trials in both the United States and Africa, but is not actively being advanced into clinical development.^6^ Currently, five vaccines are in, or planned for, clinical development – two adenovirus (Ad)-based vector vaccines and three vesicular stomatitis virus (VSV)-based vector vaccines.^6^

In this report, we summarize current insights from ongoing efforts by a World Health Organization R&D Blueprint working group on the design of randomized Phase 2b and 3 vaccine efficacy (VE) trials for candidate MARV vaccines that should soon be ready for testing. These trial designs will accommodate the complex epidemiology of MARV transmission by allowing for accumulation of evidence over multiple outbreaks that occur at different times and locations. It should be recognized, however, that such designs would not be feasible to implement unless the outbreaks that emerge are much larger than most of those documented so far. As we saw with Ebola (and of course COVID-19), it is almost impossible to know if or when such outbreaks might occur. However, it is extremely important to implement vaccine trials rapidly when infectious disease threats unexpectedly occur.^9^

## Methods

### Operating characteristics of the trial design

A core protocol approach will be used, allowing multiple vaccine candidates to be tested against a shared placebo/control group. The primary objective of the trial will be to evaluate the effect of each vaccine on the rate of virologically confirmed MARV disease, regardless of severity. In some cases, it may also be possible to evaluated MARV infection as a co-primary outcome. Although the study may lack power for formal statistical inference about VE against severe disease or death due to MARV disease, these secondary endpoints will be evaluated and reported for each vaccine. Additional exploratory endpoints will include VE between the first and second dose, for two-dose vaccines. Infecting viruses will be sequenced as much as possible to assess the influence of virus clades and evolution on transmissibility, virulence and VE. A subset of trial participants will provide periodic blood samples. Immune markers measured from participants in this immunogenicity subset will be used to assess immune correlates of risk and protection, and help with the assessment of VE against infection.

The trial is endpoint-driven, as the main analysis for each vaccine arm versus the concurrent shared placebo/control arm is triggered by the occurrence of a total of 150 cases of MARV disease across these two arms. While results from the main analysis will be reported, blinded follow-up will continue to evaluate safety and duration of immunity. This fixed number of 150 endpoints provides 90% power to rule out the hypothesis that VE is ≤ 30%, when the true VE is ≥ 60%, where statistical significance would be achieved if the estimated VE were ≥ 50%. In the setting of COVID-19 vaccines, such results would meet or exceed the World Health Organization-Food and Drug Administration (FDA) established threshold for worthwhile VE.

A global data monitoring committee will regularly review the accumulating safety results and major endpoint results. Different candidate vaccines may be available or suitable to enter the trial at different calendar times. Using a shared placebo/control group and a common core protocol to evaluate multiple candidate vaccines in the trial, resources allocated to the evaluation of each candidate vaccine will be judiciously saved while a high standard of scientific rigour and efficiency is ensured.^10^ Participants will be randomized to one of the vaccines (k in number) for which they are eligible or to one of the placebos that correspond (in appearance, dosing interval, and route of administration) to each of those vaccines. The randomization ratio will ensure that participants have the same chance of receiving a placebo (with probability 1/(k + 1) for placebo in aggregate, which is the sum of the probabilities 1/k(k + 1) for each individual placebo) as they have of receiving each individual vaccine (with probability 1/(k + 1)) for which they are eligible. Outcomes in recipients of each vaccine candidate will be compared with outcomes in all placebo recipients who were eligible to be randomized to that vaccine. This approach preserves blinding and enables comparison of each vaccine’s results directly to results from an equal number of controls who received placebo at the same time and place. Groups of sites in specific countries or subsets of sites across countries may want to collaborate in making further measurements or observations. These could be thought of as Phase 2b trials nested within the Phase 3 trial, provided that they also collect all information needed for the Phase 3 trial. Well-organized additional research studies could be conducted to assess additional secondary and supportive endpoints, such as infection or viral load (as a surrogate for transmission); nonetheless, they are not core requirements for all sites. For cluster-randomized designs, both indirect and overall vaccine effectiveness can be estimated as additional exploratory endpoints.

The trials will be double-blind to the extent possible. The best comparators will be either vaccine placebos or active comparator vaccines that are not related to protection from MARV infection, but would provide benefit to the population, for example, hepatitis A vaccine; however, a vaccine comparator could complicate safety assessments. If neither is possible, then observation-only or delayed vaccination may be used for this purpose.

### Different trial designs

**Figure 1.**
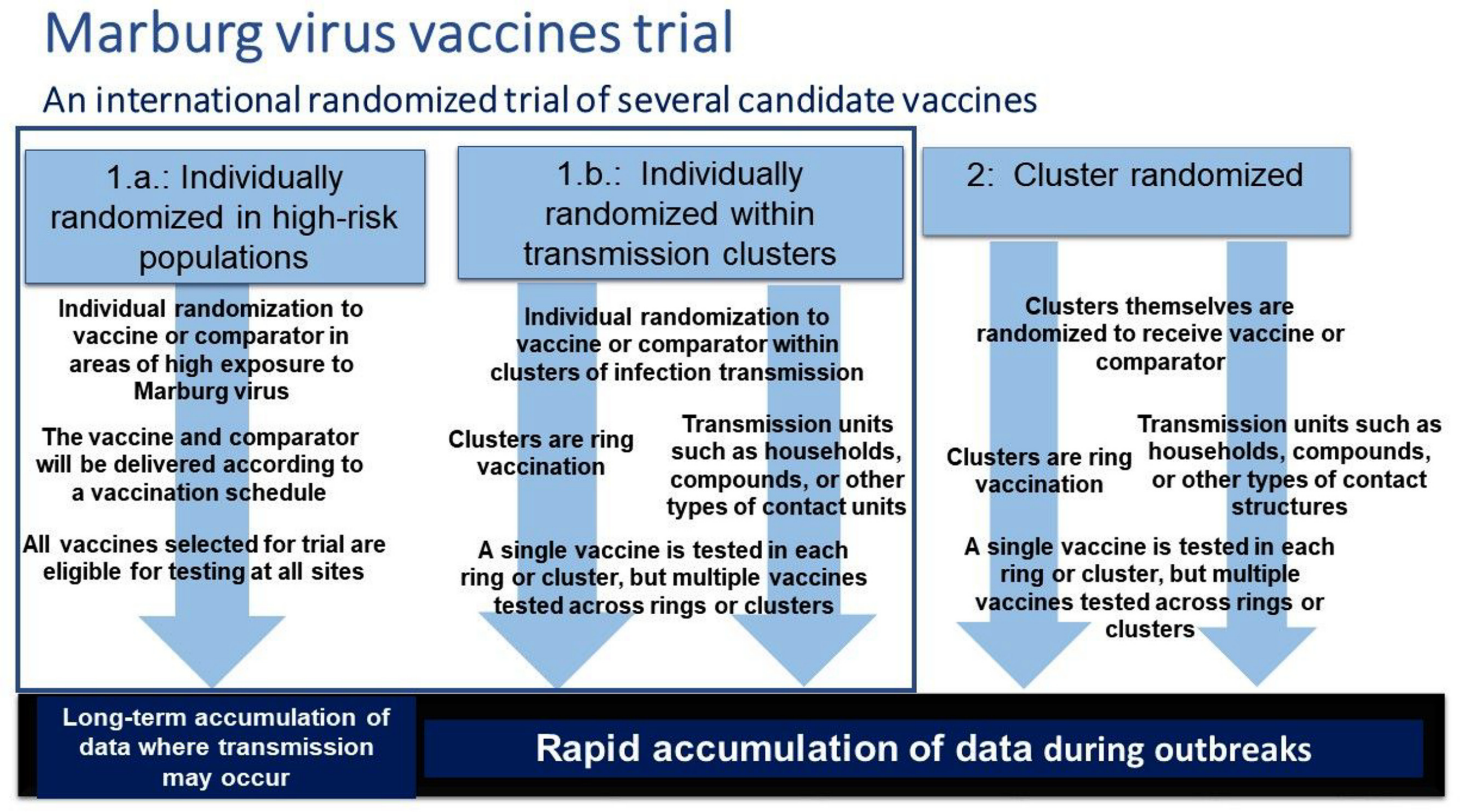
The MARV vaccine trial coordination of different designs into a platform trial with a single steering committee and data monitoring committee.

We describe two categories of possible trial designs (Figure 1).

1. Individually randomized in high-risk populations or during outbreaks
  - High-risk population
    Simple individual randomization to vaccine or placebo/control in areas of high risk of exposure to MARV, including populations living near the proposed reservoirs (e.g. miners exposed to bat caves, surrounding population where outbreak is occurring). The vaccine and comparator will be delivered according to a common vaccination schedule.
  - Transmission clusters during outbreaks
    Simple individual randomization to vaccine or comparator within clusters of infection transmission.
    - Clusters will be rings, that is, a cluster will be contacts of an index case and contacts of those contacts.
    - Clusters will be transmission units such as households, compounds, or other types of contact structures.
2. Cluster-randomized during outbreaks

Clusters themselves, defined as above in 1b (i) and 1b (ii) would be the unit of randomization; hence, all participants within a cluster would receive vaccine or all would receive the comparator.

Design category 1 is individual randomization, but design 1.a. does not involve transmission clusters, while design 1.b. involves relatively small (e.g. 100 or fewer people) clusters. Although the basic forms of statistical analysis for designs 1 and 2 are similar, there are subtle differences that need to be considered. For the individually randomized design, vaccines could be site-specific or even participant-specific. In the analysis of each vaccine, we include all participants who were randomized to either that vaccine or placebo/comparator and who were eligible for both at their randomization. The overall platform trial will be a blending of these designs in a sequential and non-overlapping way. For the cluster-randomized designs, 1.b. and 2, every effort will be made to ensure that the clusters are non-overlapping and geographically separate to avoid the potential for contamination.^11^

In some cases, it would be possible to use study design 1.a. to assess the VE against infection by recruiting cohorts of people with potential high exposure, for example, individuals having frequent contact with Rousettus bats in caves. It will take a long time to accrue events in design 1.a., but much faster for design 1.b. during outbreaks. By combining these two approaches, we will shorten the trial as much as possible. Although individual randomization is preferred, we will be able to take advantage of cluster randomization when it occurs in design 2.

## Results

### VE analysis

The primary efficacy endpoint will be analysed as a time-to-event outcome, including only those events that occur 10 days after the final vaccination dose. Such a gap of 10 days is chosen to allow time for the vaccination series to take effect and to reduce the chance of including events occurred prior to the vaccination due of the incubation period for Marburg. All participant-level failure (diagnosis with disease) and censoring events are reported relative to the time of randomization. The time of virologically confirmed (i.e. laboratory-confirmed) MARV disease is the earliest time of reported qualifying MARV disease symptoms that occur within 10 days of virological confirmation of MARV infection. Participants’ follow-up times will be right censored at the time of last contact at which endpoint status was assessed. Cox proportional hazards regression will be used for analysis. VE, measured by one minus the hazard ratio (vaccine versus placebo/comparator), will be the effect measure. Inference will be based on a one-sided logrank test of H_0_^benefit^: VE ≤ 0.30, using an O'Brien-Fleming boundary for early termination that preserves an experimental (one-sided) 0.025 error rate; the alpha levels would be one-sided 0.0003 at 50 events and one-sided 0.0069 at 100 endpoints, where the remainder of the (one-sided) 0.025 error rate would be spent at the final analysis. Using these O'Brien-Fleming criteria after 50 and 100 events, benefit is established when estimated VE is ≥80% and ≥59%, and lack of benefit, that is, ruling out the target VE of 60% for which the trial has 90% power, is established when estimated VE is ≤−6% and ≤34%, respectively. When estimating VE against infection, the outcome would be infection as measured by time to seroconversion, using interval censoring methods for the estimand. In this case, the operating characteristics of the statistics would be the same as described above.

The choice of estimand will be fully pre-specified, according to the International Council for Harmonization guidelines.^12,13^ The treatment policy estimand, based on the Intention-To-Treat principle, would provide particularly appealing properties, given its unique capability of preserving integrity of randomization, enhanced clinical relevance due to its unconditional nature, and proper evaluation of the vaccine in the context of a regimen. A variation to be considered, consistent with common practice in vaccine evaluation, would be to include only those events that occur after a specified post-vaccination period.

### Study sample size for individually randomized designs

The trial is endpoint-driven, as the primary analysis for each vaccine arm versus the concurrent shared placebo/control arm is triggered by occurrence of a total of 150 cases of MARV disease across these two arms. The primary analysis results will be reported but blinded follow-up will still continue. We assume a 1% cumulative attack rate in the control arm over 6 months of outbreak time. The trial period could involve several years of follow-up and across outbreaks. As stated above, there will be two interim analyses at the points when 1/3 and 2/3 of events have occurred, respectively. We use the O'Brien-Fleming-type spending function for both false-positive and false-negative errors. All the sample size calculations were conducted using the R package *rpact*.

For example, with a target level VE of 60%, with 150 total endpoints in a pairwise comparison, there is approximately 90% power to reject VE less than or equal to 30% if true VE is 60%, based on a logrank test with one-sided type I error rate of 0.025. With equal allocation to the active vaccine arm and the control arm, a minimum follow-up of 3 months for the last accrued participant and an annual drop rate of 10%, we calculated sample sizes for different accrual rates (Table 1). If on average 30,000 participants will be accrued each month, we need 18,062 participants per arm and an expected study duration of 9.1 months. Lower accrual rates are associated with longer study durations but smaller sample sizes. Using the sample size of 18,062 per arm, we simulated 100 times exponentially distributed event times without early stopping to investigate the coverage of the two-sided confidence interval of the VE estimate. The average total number of events is 179 and the average 95% confidence interval for the VE is (45%, 71%). The lower confidence bound would exclude 30% with a probability of 0.93, and the point VE estimate is greater than 50% with a probability of 0.91. We performed a sensitivity analysis, varying the accrual rate, assuming a 1% cumulative attack rate in the control arm over a 12-month period with an annual dropout rate of 10%, and a minimum follow-up of 6 months after the last accrual (Table 1). The sample size increases with increasing accrual rate to about 18,062 participants per arm. The best sample size is about 20,000 participants per arm, or about 40,000 participants per vaccine/comparator combination.

**Table 1.** Required total sample size, expected study duration (in months).

Accrual rate(month)	Expected study duration(months of outbreak time)	Analysis times (months ofoutbreak time)			Cumulative total number of participants(both arms)		
		First interim	Second interim	Final	First interim	Second interim	Final
1000	14.4	9.4	13.3	16.7	9,370	13,350	13,721
5000	6.6	4.2	6.0	7.8	20,771	24,248	24,248
10,000	4.9	2.9	4.4	5.9	29,292	29,292	29,292
20,000	3.8	2.1	3.4	4.7	33,873	33,873	33,873
30,000	3.4	1.8	3.0	4.2	36,124	36,124	36,124

To reduce the time to obtain reliable insights about VE, the study size should be as large as possible. The large number of sites at diverse geographical locales will smooth out uncertainty in projected MARV disease attack rates in specific locales during specific calendar time periods. To increase the reliability of results, all efforts will be made to minimize the occurrence of missing data. The occurrence of and reasons for missing data will be reported by randomization group.

In the event that design 1.a. was used to assess the VE against infection by recruiting cohorts of people with potential high exposure, but in the absence of an outbreak, then the trial outcome could be infection measured by seroconversion against MARV. This design would involve serial blood samples from trial participants, with an infection event defined as a significant rise in antibody for bracketing serum samples. Since the time of infection would be interval-censored, analysis would be carried out using survival analysis for such data.^14^ Since the primary goal of vaccination would be to prevent disease, a VE estimate based only on infection, at best, would be likely to support only an accelerated/conditional approval that would require additional post-licensure effectiveness study(ies); and at worst, it would serve only as a hypothesis-generating demonstration project.

### Study sample size for a mixture of individually and cluster-randomized designs

For those designs involving cluster randomization, the number of events needed to assess efficacy and sample sizes would be increased to account for the correlation within clusters. While the intraclass correlation coefficient (ICC) parameter for continuous and binary outcomes is commonly used, it is not easily extended to time-to-event outcome settings because of the presence of censoring (e.g. losses-to-follow-up, end-of-study administrative censoring).^15^ It has been shown that ICC estimators based on censoring indicators or observed event times can be negatively biased.^16,17^

One way to account for the clustering effect is using an ICC based on the binary event indicator and to inflate the events required by the usual design effect that equals 1 + (*m −* 1) ICC, where *m* is the average cluster size.^18^ For the Ebola ring vaccination trial in Guinea, the average ring size was about 80, and the ICC was about 0.05, resulting in a design effect of about 4. Thus, 600 total events would be needed for the design above, resulting in 150 effective events. For mixtures of individually and cluster-randomized designs, the target number of events will be the total number of effective events.

### Blending of analysis across designs

For the primary outcome, results will be combined across individually and cluster-randomized designs using a marginal proportional hazards model. We will assume that these different designs represent independent sources of evidence. Compared to the mixed-effects modelling approaches, the marginal model offers a simple marginal interpretation of intervention effects and avoids the need to specify the correlation structure among the observations. Stratified marginal models will be considered to allow differential baseline hazard functions across studies. The marginal model will be fitted under a ‘working independence’ assumption and inference will be performed using the robust sandwich variance estimator which ensures valid inference when there is a within-cluster dependence in event times.^19^ Alternative randomization-based inference methods could also be used^20^ that can account for design features (e.g. stratified randomization) in a straightforward way through restricted permutation. Another commonly used approach to combine estimates from different studies is through the use of meta-analysis approaches. When analysing censored data with a Cox proportional hazards model, the inverse-variance estimator based on the fixed-effects meta-analysis method is asymptotically equivalent to the maximum partial likelihood estimator using pooled individual-level data under a stratified model which assumes a different baseline hazard function across studies.^21^

It is important to note that the estimand for VE under individual randomization is in general different from the one under cluster randomization, with the former targeting direct vaccine effectiveness and the latter total vaccine effectiveness.^22^ (Although when direct effectiveness is high or clustering low, i.e. ICC is near zero, the two are nearly identical.) Without differentiating the two, the parameter represents a weighted average. One could parameterize the total effect as the sum of direct effects and indirect effects in the model. The availability of data from both individual and cluster-randomized trials permits the estimation of both by including an interaction term between the treatment variable and an indicator variable that takes value 1 if data are from the cluster-randomized trials and 0 otherwise.

## Conclusion

We have described a vaccine trial design for identifying an efficacious MARV vaccine as rapidly and efficiently as possible. This event-driven, platform trial design takes into account the potential sporadic spread of MARV over a wide range in Sub-Saharan Africa. The design incorporates elements of the successful ring vaccination trial structure that was employed to find an efficacious vaccine for Ebola in 2015 in Guinea.^23,24^ In that trial, a vesicular stomatitis virus-based vaccine expressing a surface glycoprotein of Zaire Ebolavirus was found to be safe and highly efficacious at the interim analysis with 90 clusters (i.e. rings), 48 with immediate vaccination and 42 with delayed vaccination, comprising a total of 7651 participants. For MARV, there have been 13 outbreaks since 1967.^25^ Most of these outbreaks have been small, with fewer than 30 cases. However, the 2005 outbreak in Angola had 374 cases, and the 1998–2000 outbreak in the Democratic Republic of the Congo totalled 154 cases. Both of these outbreaks would have passed the threshold of 150 cases needed to carry out a final analysis with the vaccine trial design described here. The timing and size of future MARV outbreaks remains uncertain, but as mentioned above, there is evidence of increased circulation of MARV in Central and Western Africa. In addition, there are a host of other potential filovirus and other infectious disease threats, with epidemic and pandemic potential, that could emerge without warning.^9^ The vaccine trial design described here could be quickly implemented should any of these threats emerge, although it could take several outbreaks to accumulate enough evidence to assess the efficacy of the vaccines deployed in the trials.

Currently, the World Health Organization COVID-19 Solidarity Vaccines trial^7^ is being deployed for the assessment of COVID-19 vaccines in multiple sites within multiple countries. This individually randomized, placebo-control trial fits roughly into category 1.a., in Figure 1. One important selection criterion for the Solidarity Vaccines trial populations has been to have an approximate 0.5%–1% COVID-19 cumulative incidence in the placebo arm over the average duration of follow-up. Other important selection criteria for sites include the ability to conduct the trial, including achieving targeted levels of enrolment and retention, and the need for more COVID-19 vaccines in the country. We propose building on the World Health Organization Solidarity trial design by adding the ability to assess the VE from transmission clusters and individual populations, to accelerate the case counts to meet the proposed endpoint totals. We allow for combining information across trial designs that involve both individual and cluster randomization. The goal would be to achieve an average 1% cumulative case incidence across the clusters. Because cluster-randomized designs require a substantially larger sample size than individually randomized trials, and because it is difficult to ensure comparability of baseline risk unless the number of clusters is very large, this design is less likely to be selected. The evaluation of performance characteristics for the blended analyses of individual and cluster-randomized would benefit from further investigation. Finally, like the World Health Organization Solidarity trial, we propose trial coordination of the different designs into a platform trial with a single steering committee and data monitoring committee. This proposed vaccine trial design can be implemented when MARV outbreaks occur, or for other emerging pathogens of high importance.
